# Supplementary figures and images for: Can audit and feedback improve health service readiness and delivery outcomes in a low-resource setting? Effectiveness results of the IDEAs strategy from central Mozambique
Source: PLOS Glob Public Health. 2025 May 12;5(5):e0004216. doi: 10.1371/journal.pgph.0004216 (PMC12068616; doi:10.1371/journal.pgph.0004216)

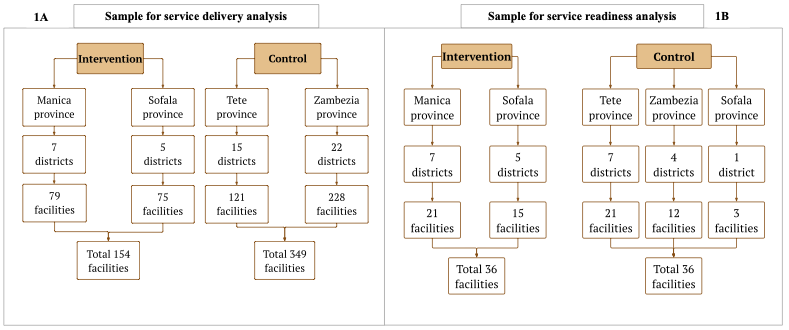

Supplement: S1 Fig — The figure is divided into part A, containing the sampling of districts and health facilities used to assess service delivery analysis, and part B, with the subsampling for service readiness outcomes. (TIFF) [file pgph.0004216.s001.tiff]
