# Supplementary material for: Can audit and feedback improve health service readiness and delivery outcomes in a low-resource setting? Effectiveness results of the IDEAs strategy from central Mozambique
Source: PLOS Glob Public Health. 2025 May 12;5(5):e0004216. doi: 10.1371/journal.pgph.0004216 (PMC12068616; doi:10.1371/journal.pgph.0004216)
Supplement: S1 Appendix — It presents four different lists of items selected from the service availability and readiness survey to create composite scores for readiness outcomes: the availability of essential medicine, essential infrastructure, essential services provided, and essential equipment. (DOCX) [file pgph.0004216.s002.docx]

| **List of items included to create composite scores for availability of essential medicines** |
| --- |
| 1. Antibiotics: Gentamicin, ampicillin, metronidazole, benzathine benzylpenicillin, procaine benzylpenicillin, ceftriaxone, cefixime, amoxicillin(infantile) 2. Antihypertensives: Hydralazine, methyldopa 3. Corticosteroids: Dexamethasone, hydrocortisone 4. Intravenous rehydration solution: Sodium chloride, sodium lactate compound, glucose 5% compound 5. Antimalarials: Artesunate, artemisinin combination therapy (ACT), quinine 6. Oxytocin 7. Magnesium sulfate 8. Nifedipine 9. Misoprostol 10. Vitamin A (infant) 11. Calcium gluconate 12. Oral rehydration salts (ORS) 13. Zinc 14. Oxygen 15. Tetanus vaccine |
| **List of items included to create composite scores for availability of infrastructure** |
| 1. Communications: Functioning landline telephone, cellular, short-wave radio, computer 2. Transportation: Functional ambulance or other vehicles for emergencies, fuel for the emergency vehicle 3. Power supply: Electricity from any source, secondary backup source 4. Basic amenities: Functioning toilet for outpatients, availability of water in the main source, privacy available for patients 5. Processing of equipment for reuse: Electric autoclave, electric boiler or steamer, non-electric pot with cover for boiling/stem, a heat source for non-electric equipment 6. Infection control: Cleaning running water, hand washer, alcohol-based hand rub, disposable latex gloves, waste receptacle with lid and plastic bin liner, sharps container, environmental disinfectant, disposable syringes with disposable needles, auto-disables syringes, guidelines for infection control |
| **List of items included to create a composite score for essential services provided** |
| 1. Monitoring for a hypertensive disorder of pregnancy 2. Provide ARV prophylaxis to HIV-positive pregnant women for PMTCT 3. Monitoring and management of labor using partograph 4. Administration of oxytocin injection immediately after birth to all women for the prevention of post-partum hemorrhage 5. Parenteral administration of oxytocic for treatment of post-partum hemorrhage (IV or IM) 6. Hygienic cord care (cut with a sterile item and apply disinfectant to tip and stump, and no application of other substances) 7. Parenteral administration of antibiotics (IV or IM) for mothers 8. Antibiotics for preterm or prolonged PROM (premature rupture of membranes) to prevent infection 9. Parenteral administration of magnesium sulfate for management of preeclampsia and eclampsia (IV or IM) 10. Injectable antibiotics for neonatal sepsis 11. Corticosteroids in preterm labor 12. Immediate and exclusive breastfeeding 13. Thermal protection (drying baby immediately after birth and wrapping) 14. Neonatal resuscitation with bag and mask 15. KMC (Kangaroo mother care) for premature/very small babies 16. Provide ARV prophylaxis to newborns of HIV-positive pregnant women for PMTCT |
| **List of items included to create a composite score for the availability of essential equipment** |
| 1. Disposable latex gloves 2. Birth kit 3. Incubator 4. Infant weighing scale 5. Sphygmomanometer 6. Electric suction pump 7. Suction catheter for newborn 8. Resuscitation table for newborn 9. Newborn bag and mask size 1 for term babies 10. Newborn bag and mask size 10 for pre-term babies 11. Oxygen delivery apparatus (key connecting tubes and mask/nasal prongs) 12. Oxygen bottles 13. Manual vacuum extractor 14. Partogram 15. Speculum 16. Thermometer 17. Rapid diagnostic test, HIV 18. Rapid diagnostic test, malaria 19. Rapid diagnostic test, syphilis 20. Syringes 21. Suture material with needles available 22. Hemoglobin color scale 23. Blood glucometer 24. Umbilical clamp 25. Intravenous kit 26. Examination light 27. Vacuum aspirator or D&C kit 28. Pinard stethoscope |
