# Supplementary material for: Can audit and feedback improve health service readiness and delivery outcomes in a low-resource setting? Effectiveness results of the IDEAs strategy from central Mozambique
Source: PLOS Glob Public Health. 2025 May 12;5(5):e0004216. doi: 10.1371/journal.pgph.0004216 (PMC12068616; doi:10.1371/journal.pgph.0004216)
Supplement: S1 Table — This table presents the characteristics of the baseline unmatched and matched data via propensity score. The variables displayed include the number of maternal and child nurses, the number of facility beds, type of facility, location, distance from the health department, distance from referral health facility, and population catchment area size. (DOCX) [file pgph.0004216.s003.docx]

| **Variables**  **Mean(SD)** | **Unmatched Baseline Data** | | | **Matched Baseline Data** | | |
| --- | --- | --- | --- | --- | --- | --- |
|  | **Control (N= 349)** | **Intervention (N=154)** | **Overall (N= 503)** | **Control (N= 300)** | **Intervention (N=150)** | **Overall (N=450)** |
| MCH Personnel | 2.46(4.44) | 3.29(5.30) | 2.71(4.73) | 2.61(4.71) | 3.29(5.30) | 2.84(4.92) |
| Facility beds | 7.19(12.5) | 7.71(15.1) | 7.35(13.3) | 7.52(13.1) | 7.89(15.3) | 7.64(13.9) |
| Type of HF | | | | | | |
| Rural Center | 307(87.9%) | 117(76.0%) | 424(84.3%) | 261(87.0%) | 114(76.0%) | 375(83.3%) |
| Urban Center | 28(8.0%) | 22(14.3%) | 50(10.0%) | 26(8.7%) | 21(14.0%) | 47(10.4%) |
| District Hospital | 4(1.1%) | 3(1.9%) | 7(1.4%) | 3(1.0%) | 3 (2.0%) | 6(1.3%) |
| Rural Hospital | 4(1.1%) | 2(1.3%) | 6(1.2%) | 4(1.3%) | 2(1.3%) | 6(1.3%) |
| Health Post | 6(1.7%) | 10(6.5%) | 16(3.2%) | 6(2.0%) | 10(6.7%) | 16(3.6%) |
| Location | | | | | | |
| Rural | 320(91.7%) | 128(83.1%) | 448(89.0%) | 273(91.0%) | 125(83.3%) | 398(88.4%) |
| Urban | 29(8.3%) | 26(16.9%) | 55(11%) | 27(9.0%) | 25(16.7%) | 52(11.6%) |
| Distance From Province Health Department (Km) | 240  (152) | 118  ( 89.1) | 203  (147) | 206  (125) | 118  (89.1) | 177  (121) |
| Distance From Referral HF(Km) | 59.6(66.1) | 41.0(39.7) | 54.0(60.0) | 53.8(57.1) | 41.0(39.7) | 49.5(52.3) |
| Catchment Area Size (Thousands Population) | 37300  (44900) | 34200  (38800) | 36400  (43100) | 36600  (44500) | 34300  ( 39300) | 35800  (428000) |
| MCH: Maternal and child; HF: Health facility; SD: Standard deviation | | | | | | |

**Supplemental Table: Baseline characteristics of study facilities in unmatched and matched data**
