# Supplementary material for: Can audit and feedback improve health service readiness and delivery outcomes in a low-resource setting? Effectiveness results of the IDEAs strategy from central Mozambique
Source: PLOS Glob Public Health. 2025 May 12;5(5):e0004216. doi: 10.1371/journal.pgph.0004216 (PMC12068616; doi:10.1371/journal.pgph.0004216)
Supplement: Attachment — Submitted filename: Response to Reviewers.pdf [file pgph.0004216.s005.pdf]

Response to Reviewers

We thank the editors and reviewers for reading and providing constructive critiques and complements to our work. Your feedback was well received, helped us improve the manuscript, and encouraged us to continue working on testing, evaluation, and dissemination of health interventions.

Below each question or comment, we use **R:** for a response in blue. Some excerpts from the manuscript are in red. The reference lines in our response are from the revised manuscript.

Editors' comments

1. Please amend your detailed Financial Disclosure statement. This is published with the article. It must therefore be completed in full sentences and contain the exact wording you wish to be published.

   R: We amended the financial disclosure statement by clarifying the funding sources, stating the initial of each author, the funding source and purpose, and the role of the funders (lines 582-587).

2. In the online submission form, you indicated that "The data supporting this study's findings are available upon reasonable request from the corresponding author and with permission of the Ministry of Health of Mozambique and Manica and Sofala provincial health directorates."

   R: we revised the statement in the manuscript. Now it reads:

   The datasets for analyzing the impact on readiness outcomes have been uploaded to a public repository and can be assessed without restrictions: https://doi.org/10.7910/DVN/CVUIAX. The data for analyzing the impact on service delivery outcomes is non-public, governmental. The minimal dataset for this data can be sourced from the custodian of the data (www.misau.gov.mz) upon reasonable request.

3. Please provide an Author Summary

   R: We added an author summary (lines 72-84).

No changes were made in the references.

Reviewer's Responses to Questions

1. Does this manuscript meet PLOS Global Public Health's publication criteria? Is the manuscript technically sound, and do the data support the conclusions? The manuscript must describe methodologically and ethically rigorous research with conclusions that are appropriately drawn based on the data presented.

Reviewer #1: Yes

Reviewer #2: Yes

2. Has the statistical analysis been performed appropriately and rigorously?

Reviewer #1: I don't know

Reviewer #2: I don't know

3. Have the authors made all data underlying the findings in their manuscript fully available (please refer to the Data Availability Statement at the start of the manuscript PDF file)?

The PLOS Data policy requires authors to make all data underlying the findings described in their manuscript fully available without restriction, with rare exception. The data should be provided as part of the manuscript or its supporting information, or deposited to a public repository. For example, in addition to summary statistics, the data points behind means, medians and variance measures should be available. If there are restrictions on publicly sharing data—e.g. participant privacy or use of data from a third party—those must be specified.

Reviewer #1: Yes

Reviewer #2: Yes

4. Is the manuscript presented in an intelligible fashion and written in standard English?

PLOS Global Public Health does not copyedit accepted manuscripts, so the language in

submitted articles must be clear, correct, and unambiguous. Any typographical or grammatical errors should be corrected at revision, so please note any specific errors here.
Reviewer #1: Yes
Reviewer #2: Yes

R: Thank you for your observation and positive feedback on the points 1-4 above.

5. Review Comments to the Author

Reviewer #1: General points

This is an impressive pragmatic intervention study to evaluate the impact of an audit and feedback intervention on maternal and child health service provision in Mozambique.
R: Thank you for your observation and positive feedback.
I think some delineation between the two studies is needed – especially in the methods and results sections. I found it a little hard to follow at times which study (readiness focused or outcomes-focused) which subsection was referring to. It may be that a simple labeling of Study 1 and Study 2 are needed throughout the paper.
R: Thank you for your observation. To clarify, these are not two studies. It's just one study with two sets of outcomes: 1)service delivery outcomes and 2)service readiness outcomes. Because of the nature of these outcomes(service delivery outcomes are monthly counts and service readiness are mostly proportions), we conducted separate statistical analyses to account for it. To clarify this point, we changed the wording in the study design (lines 206-209) and statistical analysis sections (lines 303 and 351).

Also, I wonder conceptually about 'service readiness' precluding the delivery of an intervention. Should one assess service readiness before deciding to implement something. This could be done as a 'screening' process to identify needs before implementation a program. If a service is not 'ready' then is it reasonable to think that we can make significant changes and to maintain over time. Plus, it allows the

opportunity to match gaps in readiness with strategies to support implementation. I wonder whether the authors had any thoughts about this and if so, could perhaps integrate this into the discussion.

R: This is a good point. We added a sentence in the manuscript to define service readiness to avoid confusion(lines 150-152), and please read our argument to your point as follows:

The service readiness we are examining is related to the availability of structural resources. Usually, this readiness is measured using a survey tool, and in our context, the WHO Service Availability and Readiness Assessment (SARA) survey was used. Availability and readiness in the SARA context means the existence and functionality of a commodity. SARA can be used in the initial phase of an intervention to set baselines, during the implementation, or to help manage a health system in any period. In other words, the tool's objective is to generate reliable and regular information on service delivery, but the timing of its use depends on the research question or implementation needs. In our case, the objective was to use this information to evaluate improvements in availability over time that could be associated with implementing the IDEAs strategy. More information about SARA can be found here.

In implementation science, we have **organizational assessment and readiness**, a more comprehensive concept for evaluating the degree to which an organization is prepared or ready to implement something new. In this case, the assessment is made before implementation and comprises not only the availability of structural resources but also providers' perspectives, capacity, commitment, and motivation to change practice. We think this is more aligned with your comment and is not the focus of our manuscript. More information on organizational readiness can be found here.

Reviewer #1: Specific points

L49 – 'a multi-component audit and feedback strategy' - Perhaps a sentence summarizing the components within the intervention

R: Thank you for your suggestion. We agree. To accommodate this point, we changed the abstract content slightly and added this information (lines 44-45).

L57 - Incidence rate ratios?

R: Yes. We added the missing “rate”(lines 51-52).

L61 – Define PCR?

R: Agreed. We change to “polymerase chain reaction”(line 56).

L69 – ‘IDEAs were associated with improvements in selected indicators from child-at-risk services and available infrastructure’ - This seems selective given you report multiple positive associations in the results section. Perhaps drop or as in 'such as child-at-risk services’ etc.

R: Thank you for your suggestion. We removed the selective statement and changed the wording to improve understanding (lines 64-66).

L88 – state that the United Nations lead SDG program.

R: Thank you for your suggestion. We accommodate it by adding a statement indicating that the United Nations leads the SDGs (line 101).

L138 - Does ‘service readiness’ as a term perhaps need to be defined here or beforehand in the intro.

R: We agree. We added the definition of service readiness to facilitate understanding (lines 150-152).

L144 – ‘address multi-level barriers and facilitators for delivery’ - How was this in particular operationalized? No real mention of barriers and enablers assessment in the paper. Is it referring to the readiness assessment? More clarity needed.

R: We will include the complete original sentence here to clarify this point: *“The IDEAs strategy is designed to improve health service delivery using A&F tools that*

identify service gaps, address multi-level barriers and facilitators for delivery, and enable health workers to prioritize and test interventions to improve the application of Ministry of Health (MOH) guidelines for neonatal health."

In the sentence above, we are describing the general concept of the IDEAs strategy, how IDEAs use A&F tools(the readiness assessment data, action plans, auditing data from routine health information system, and supervision visits assessments) to help identify gaps and address problems and solutions that can be multilevel (not only factors specific to the health facility level but also from the district or national level) enabling the MCH managers to prioritize those problems that they can solve by implementing micro-interventions. In the program description section, we expose the steps needed for this process to occur (lines 163-171), and in lines 175-198, we describe in detail these steps, including the use of tools to facilitate this purpose.

In other words, the present paper does not assess barriers and facilitators as you noted; we are just explaining that the tools used in the IDEAs strategy help to identify them.

As a side note, we have already published a manuscript on determinants (barriers and facilitators) of IDEAs implementations, which can be found here.

L162 – 'Step 2: Audit and feedback meeting' - You cite the Ivers 'no more business as usual' A&F paper which suggests several recommendations for developing A&F interventions. I wonder if it might be informative to the reader to map your A&F intervention to some of the features reported in the Ivers paper. I think this could speak to the point around the 'refinement' of the intervention which you mention in the discussion. So, which bits of the A&F intervention worked well, where are their opportunities to enhance the reports, can we build more theory into the A&F intervention etc.

R: This is a great comment, and we have answered it with the following arguments:

In line you mention (previously line 162, but in the revised paper is line 180), we describe the steps of the IDEAs strategy design, and we did not cite Ivers in this description. However, to your point, IDEAs strategy design incorporates multiple aspects described by Iver's et al. For instance, the characteristics and components of IDEAs(Maternal and child health managers as leading actors, use of data to inform

service delivery and readiness, the cycle nature of the A&F meetings, and the elaboration and use of action plans) characterize the best practices for A&F design suggested by Iver's at al. We added a sentence to emphasize the use of best practices in IDEAs design (lines 172-173).

We tested this implementation strategy to inform a potential scale-up, and the adaptations(or refining) aspects need to be contextualized to our findings. By incorporating best practices in the strategy's design, embedding this research in a real-world setting, and drawing context-specific conclusions for strategy improvement, we are well aligned with the discussion presented by Ivers et al.

A piece of side information that might help you understand our work: the present manuscript is just one of a series of papers prepared to share the learnings from IDEAs A&F strategy. We published the first paper explaining the implementation process and detailed information on what went wrong, including identifying the key `active ingredients` of the strategy mentioned by Ivers et al. More information can be found here. The present paper focuses only on reporting the effectiveness of IDEAs on service outcomes. A third forthcoming paper studies the link between effectiveness and fidelity of implementation. In other words, we examine the link between the key ingredients of the strategy and the effectiveness results. This body of work tries to respond to at least two of the points discussed in Iver's paper: that little progress has been made in identifying the key ingredients for A&F intervention in healthcare and that it is imperative to improve our understanding of when and why an A&F achieves its effects.

We added more information on the study design section to pinpoint that this manuscript is not an isolated analysis we use to draw conclusions about the strategy.

L169 – what do you mean by micro-interventions?

R: We defined micro-interventions in line 165-167 and improved the wording for better understanding. It reads: "A&F meetings to identify gaps and develop action plans(comprised of priority problems and *micro-interventions* defined as solutions identified, specified and implemented at the health facility level." We also added examples of micro-interventions in lines 187-190.

L170 – ‘Step 3: Targeted facility support.” – Out of interest, are there are data on how these selected facilities fared in terms of outcomes? How did they compare to those that did not receive additional support. Did their selection lead to marked improvements in the low-performing facilities?

R: This is a great question. The categorization of high and low-performing was made based on the performance of service indicators. In each A&F meeting, each facility presented the status of key indicators, and the maternal and child managers (participants of the meetings) used their judgment to evaluate which facilities were poor or good performers. The goal of this exercise was to select facilities that needed target supervision. In evaluating the implementation process, we observed that 321 selections(214 low-performing and 107 high-performing) were made over the implementation period. The majority, 83% of all 154 health facilities, had been selected at least once. The average number of supervision visits was higher for low-performing facilities than for high-performing ones (3.7 versus 2.6 times). However, it’s not adequate to make discrete comparisons of improvements because the same facility could be selected multiple times across the A&F meetings rounds and sometimes switch from high to low-performing categories or vice versa. More about the strategy implementation process can be found here.

L212 – ‘(totaling 36 intervention and 36 control facilities)’ - Are these facilities a subset of the 154 intervention and 349 control facilities? If so, would make this clear.

R: Good observation and suggestion. All 36 intervention facilities are a subset of the 154. Thirty-three control facilities are a subset of the 349. Three additional facilities from one district of Sofala were added to the control subset. We added a sentence with this explanation(lines 238-241).

L228 – use acronym A&F

R: Thank you for your observation. It is done(line 257).

L365 - How were the exploratory variables selected? What was the criteria for judging their relevance.

R: To select explanatory variables, we applied knowledge of identifying and selecting confounders. We verify 1) if the variable is associated with the outcome, 2) if it is unequally distributed between the groups we are comparing, and 3) if it is not an intervention effect. Among a long list of these variables, the inclusion or not in the regression model mainly depended on the alignment of the variable with the IDEAs theory of change, research question, and data availability.

L404 – Opening sentence of discussion too long, would suggest rewriting.

R: Thank you for the suggestion. We rewrote the opening sentence(lines 433-436).

L417 – Not sure what 'robust vertical resources' means, perhaps define.

R: Good observation. We changed the writing to be more precise (lines 445-447). Now it reads: *"...improvements in at-risk child services outcomes may highlight a more solid availability of resources in the verticalized HIV services compared with other services…"*

L421 – again more clarity about the micro-interventions needed

R: Agreed. The precise definition and examples are presented in lines 165 -166 and 187-190, respectively.

L458 - Mentioned that this study was an overview of systematic reviews.

R: Correct, it is an overview of systematic reviews. We have clarified in the text (line 488).

L528 – 'refining and adapting strategy components to target clinical and readiness outcomes more directly' – really like this point but could you posit how you might approach this.

R: this extract of the text is from the conclusion section. Before that section, we explained what we meant by refining and adapting the strategy. In lines 540-546, we recommend allocating supporting funding at the facility level instead of the district level as initially designed and changing the supervision approach in focusing support around managing obstetric care. Moreover, adaptation can also include combining A&F with other strategies to maximize potentiality in impacting service outcomes – this is also posited in lines 552-554, in which we give the example of combining A&F with training of MCH nurses in managing emergency obstetric care.

Reviewer #2: OVERALL

This is a well-written paper concerning the use of a recognized healthcare quality improvement technique applied in a new context. Unfortunately, my understanding of statistics is not sophisticated, so I cannot comment extensively on the statistical methods used. However, the presentation of incidence ratios for service delivery outcomes and odds ratios for service readiness outcomes seems reasonable as both are relatively straightforward to interpret for a range of skill levels.

R: Thank you for your observation and positive feedback

TITLE

The Title adequately indicates the topic addressed and setting for this study.

R: Thank you for your feedback.

ABSTRACT

The Abstract provides a good summary of the article.

R: Thank you for your feedback.

INTRODUCTION

The Introduction is very informative about the context of the study and clearly emphasises the importance of finding ways to combat the major causes of neonatal mortality in central Mozambique.

R: Thank you for your feedback.

METHODS

The Methods are clearly described and in a good level of detail. The details contained in Appendices/Supporting Information is very helpful.

R: Thank you for your feedback.

RESULTS

The Results are well-presented, with a good balance between narrative and graphical information.

R: Thank you for your feedback.

DISCUSSION

The Discussion is well-written and reflects the aims and findings of the study. The authors clearly identify and acknowledge the strengths of the paper and offer honest explanations of any weaknesses. Their conclusions are reasonable and well-expressed. The authors have included some recommendations within the Discussion section – it may be helpful for these recommendations to be in a separate section.

R: Thank you for your suggestion. We added a subsection for recommendations and future steps (Lines 532-556).

REFERENCES

The references comprise a good range of topic areas and years of publication.

R: Thank you for your observation and positive feedback.
