# Supplementary material for: Can audit and feedback improve health service readiness and delivery outcomes in a low-resource setting? Effectiveness results of the IDEAs strategy from central Mozambique
Source: PLOS Glob Public Health. 2025 May 12;5(5):e0004216. doi: 10.1371/journal.pgph.0004216 (PMC12068616; doi:10.1371/journal.pgph.0004216)
Supplement: Attachment — Submitted filename: Response_to_Reviewers_auresp_2.pdf [file pgph.0004216.s007.pdf]

Thank you for reviewing our work and for providing constructive feedback that improves this manuscript. We hope we satisfactorily addressed all your questions. Our responses are in blue, and the line numbers mentioned are based on the track-change document sent

This study presents original research using a quasi-experimental study design to investigate the effect of audit and feedback. The article is well written with very minimal grammatical errors. Appropriate statistical analysis was done using controlled time series and a difference in difference in difference analysis. Expected ethical guidelines were considered.

R: Thank you for your positive feedback

The study had several limitations which may have contributed to the lack of association noted , however the authors discussed these limitations. Furthermore the study lacked a qualitative component which could have provided more insight about the negative associations or lack of associations. The authors could consider recommending the inclusion of a qualitative component in future research about A and F.

R: We agree with your observations. Implementing research in real-world settings presents many challenges, but it also provides a more realistic understanding of the complexity of health systems. A qualitative evaluation of this intervention was conducted initially and is published **here**. We want to take this opportunity to inform the reviewer that this is not an isolated analysis; rather, it is part of a series of manuscripts submitted regarding the IDEAs strategy. For example, we evaluated the implementation process and reported implementation outcomes (manuscript published **here**); the current manuscript focuses solely on the effectiveness of service delivery and readiness outcomes, while a forthcoming publication will address the relationship between effectiveness and fidelity of implementation. Through all this work, we aim to explain in various ways how the implementation occurred, what results were observed, and the reasons behind these results.

Clarify whether the service delivery outcomes were compared across similar facilities in the intervention and the control. The clarification should be done by providing more detail about the level and types of facilities that were included in the intervention and the control arm ( Line 202 – 207).

R: Thank you for your recommendation. To clarify this point, all these are facilities that provide primary health care services within the public health sector. They carry out the same maternal and health activities and maintain the same indicators recorded in the routine health information system, which is the source of our data. The types of facilities include rural and urban primary health care centers (93.1%), health posts (4.1%), and rural and district hospitals with primary health care services (2.8%). We have added information about the level and types of the control and intervention facilities in lines 230-232.

The authors attempted to match the facilities between the intervention and the control, however differences still persisted as noted in this sentence (Line 343 -345) "*Most facilities are rural health centers located, on average, more than 50 kilometers from the referral health facility. Intervention facilities tend to have a slightly higher number of MCH nurses, include more health posts, and are more likely to be located in an urban center than controls*" Can the authors comment on the effect of these persistent differences on their results. This issue was not adequately discussed in the discussion section.

This is a great observation. Indeed, we did our best to match well the facilities. However, we believe that the remaining differences reflect the reality of the distribution of health facilities in the country. 95.9% of health facilities in Mozambique are primary-level and 84.4% of them are located in rural areas. However, because the matching was not perfect, we have included covariates (location, type of facility, Covid period, province GDP, facility population catchment area size) in our models to minimize further confounding. Given the lack of randomization, that was the best we could do. We added some information in lines 347-348 and 518-520 to respond to your point.

The explanations provided about the null effects largely do not acknowledge the influence of demand side factors that often influence the utilization of MCH services and that require significant resources  and multisectoral efforts to address. This should be added to the discussion .

R: We agree with you that demand factors are important determinants of utilization of MCH services. However, based on IDEAs strategy design, our experience implementing this intervention in this setting, and other analyses we are conducting regarding this strategy, we are confident that demand was not an important influencer for the majority of null results. We believe that weakness in implementing activities of the action plans ( either due to lack of resources or poor training of MCH nurses) and untailored supervision were the major factors. For family planning indicators, we agree that demand might play a role because of the reduction in  service provision due to external factors, and we discussed that in lines 472-483.

In the conclusion section , the authors report that "*Sub-optimal and null effects in most indicators suggest a need for refining and adapting strategy components to target clinical and readiness outcomes more directly in a low resource context.*" I find this conclusion rather vague. Additionally  in their discussion they seem to suggest that the null effects were most likely due to inadequate resources for addressing the challenges identified, they could also reflect the complexity associated with the interventions, including demand side constraints   . I therefore recommend that they revise this second part of the conclusion.

R: Thank you for your observation. We added some information in discussion (lines 451-452) and edited the conclusion to reduce vagueness and include a reflection on intervention complexity. Now it reads:

"Suboptimal and null effects across most indicators suggest that various health system factors enhance the complexity of implementing the intervention in this context, indicating the need for adjustments in the strategy design, implementation and evaluation to maximize the impact. Therefore, we recommend refining and adapting IDEAs strategy components to target more directly clinical and readiness outcomes."
