# Supplementary material for: Can audit and feedback improve health service readiness and delivery outcomes in a low-resource setting? Effectiveness results of the IDEAs strategy from central Mozambique
Source: PLOS Glob Public Health. 2025 May 12;5(5):e0004216. doi: 10.1371/journal.pgph.0004216 (PMC12068616; doi:10.1371/journal.pgph.0004216)
Supplement: Attachment — Submitted filename: Mozambique study.docx [file pgph.0004216.s006.docx]

This study presents original research using a quasi-experimental study design to investigate the effect of audit and feedback. The article is well written with very minimal grammatical errors. Appropriate statistical analysis was done using controlled time series and a difference in difference in difference analysis. Expected ethical guidelines were considered.

The study had several limitations which may have contributed to the lack of association noted , however the authors discussed these limitations. Furthermore the study lacked a qualitative component which could have provided more insight about the negative associations or lack of associations. The authors could consider recommending the inclusion of a qualitative component in future research about A and F.

Clarify whether the service delivery outcomes were compared across similar facilities in the intervention and the control. The clarification should be done by providing more detail about the level and types of facilities that were included in the intervention and the control arm ( Line 202 – 207).

The authors attempted to match the facilities between the intervention and the control, however differences still persisted as noted in this sentence (Line 343 -345) “*Most facilities are rural health centers located, on average, more than 50 kilometers from the referral health facility. Intervention facilities tend to have a slightly higher number of MCH nurses, include more health posts, and are more likely to be located in an urban center than controls*” Can the authors comment on the effect of these persistent differences on their results. This issue was not adequately discussed in the discussion section.

The explanations provided about the null effects largely do not acknowledge the influence of demand side factors that often influence the utilization of MCH services and that require significant resources and multisectoral efforts to address. This should be added to the discussion .

In the conclusion section , the authors report that “*Sub-optimal and null effects in most indicators suggest a need for refining and adapting strategy components to target clinical and readiness outcomes more directly in a low resource context.*” I find this conclusion rather vague. Additionally in their discussion they seem to suggest that the null effects were most likely due to inadequate resources for addressing the challenges identified, they could also reflect the complexity associated with the interventions, including demand side constraints . I therefore recommend that they revise this second part of the conclusion.
